# Retraction and replacement of: The relationship between sexual dimorphism and androgen response element proliferation in primate genomes

**DOI:** 10.1093/evolut/qpad182

**Published:** 2023-11-24

This is a retraction and replacement of: Andrew P. Anderson, Adam G. Jones, The relationship between sexual dimorphism and androgen response element proliferation in primate genomes, *Evolution*, Volume 76, Issue 6, 1 June 2022, Pages 1331–1346, https://doi.org/10.1111/evo.14483

On 01 June 2022, *Evolution* published an article entitled ‘The relationship between sexual dimorphism and androgen response element proliferation in primate genomes’ by Andrew P. Anderson and Adam G. Jones.

On 30 June 2023, the authors contacted the journal about an error that had been discovered in the R code used in their article, which changes the data presented in Table 1 and which would affect the interpretation of the results of some of the analyses presented. Regarding the specific nature of the error, the calculation of correlation values between the Androgen Response Elements (AREs) and indicators of sexual selection did not appropriately match tree tip labels with the data set. Given the putative changes needed in the main text, tables and figures were deemed beyond the scope of a correction notice, the authors and editors agreed to a retraction and republication to correct the record, following a thorough reanalysis.

In the recalculation of correlation values, the authors observed a few models (*n* = 6 out of 57 models) with estimated lambdas much greater than one or much less than 0 (lambda estimates were restricted to between 0 and 1) and report both these new findings and all correlation values in the amended Table 1. The new correlation values no longer show any meaningful over-representation of positive correlations for relative testis size or canine sexual size dimorphism, but positive correlations tend to be of greater absolute values than negative values. Some specific AREs also display significant correlations with canine sexual size dimorphism or testis size, and these significant correlations are all positive. The restricted models cause a loss of significant correlations with motif counts of *psa3* with both body mass sexual dimorphism and relative testis size, *cSRE* with canine size sexual dimorphism, and the principal component of androgen response elements (PC ARE) with relative testis size (Table 1). Following this and using the corrected correlation values, the authors re-performed the comparative analysis of the effects of AREs on sex bias and expression (Table 2), the multivariate analysis (Figure 3) and the haplotype network analysis (Figure 4), which resulted in changes in some of the interpretations of these analyses. The reanalysis can be found at https://github.com/AndersonDrew/Primate_ARE.

Following this reanalysis, the overall findings of strong evidence of influence of canine size sexual dimorphism on genome-wide androgen response elements are still supported. The claim of an influence of testis size was primarily based on evidence from the number of positive correlations, a significant correlation with PC ARE, and the output of the canonical correlation analysis (CCorA). The CCorA, a positive correlation with genome-wide counts of the *hklk* motif, and overall greater positive correlation values still provide some weak evidence of a positive relationship between relative testis size and genome-wide ARE counts. The authors also originally reported no effect of body mass sexual dimorphism on genome-wide ARE counts, a pattern that is still present in the new analysis. However, they did observe a large number of negative correlations between individual ARE counts and body mass sexual dimorphism, and the CCorA has body mass as largely positive on the first dimension, with most ARE counts loading negatively, suggesting a possible inverse correlation.

This retraction is to clarify the scholarly record and in no way suggests any misconduct on the part of the authors. The editors commend the authors for bringing the error to the journal’s attention and for their desire to present an accurate account of their results.

The new version of the manuscript was reviewed and accepted by the handling editors at *Evolution* and has been republished at https://doi.org/10.1093/evolut/qpad181.

